# Dominantly inherited myosin IIa myopathy caused by aberrant splicing of *MYH2*

**DOI:** 10.1186/s12883-022-02935-4

**Published:** 2022-11-15

**Authors:** Carola Hedberg-Oldfors, Ólöf Elíasdóttir, Mats Geijer, Christopher Lindberg, Anders Oldfors

**Affiliations:** 1 Department of Laboratory Medicine, Institute of Biomedicine, Sahlgrenska Academy, University of Gothenburg, Gothenburg, Sweden; 2 Neuromuscular Center, Department of Neurology, Sahlgrenska University Hospital, Gothenburg, Sweden; 3 Department of Radiology, Institute of Clinical Sciences, Sahlgrenska Academy, University of Gothenburg, Gothenburg, Sweden; 4 Department of Radiology, Region Västra Götaland, Sahlgrenska University Hospital, Gothenburg, Sweden; 5 Department of Clinical Sciences, Lund University, Lund, Sweden

**Keywords:** MYH2, Autosomal dominant, Myopathy, Myosin heavy chain, Splice-site

## Abstract

**Background:**

Myosin heavy chain (MyHC) isoforms define the three major muscle fiber types in human extremity muscles. Slow beta/cardiac MyHC (*MYH7*) is expressed in type 1 muscle fibers. MyHC IIa (*MYH2*) and MyHC IIx (*MYH1*) are expressed in type 2A and 2B fibers, respectively. Whereas recessive MyHC IIa myopathy has been described in many cases, myopathy caused by dominant *MYH2* variants is rare and has been described with clinical manifestations and muscle pathology in only one family and two sporadic cases.

**Methods:**

We investigated three patients from one family with a dominantly inherited myopathy by clinical investigation, whole-genome sequencing, muscle biopsy, and magnetic resonance imaging (MRI).

**Results:**

Three siblings, one woman and two men now 54, 56 and 66 years old, had experienced muscle weakness initially affecting the lower limbs from young adulthood. They have now generalized proximal muscle weakness affecting ambulation, but no ophthalmoplegia. Whole-genome sequencing identified a heterozygous *MYH2* variant, segregating with the disease in the three affected individuals: c.5673 + 1G > C. Analysis of cDNA confirmed the predicted splicing defect with skipping of exon 39 and loss of residues 1860–1891 in the distal tail of the MyHC IIa, largely overlapping with the filament assembly region (aa1877–1905). Muscle biopsy in two of the affected individuals showed prominent type 1 muscle fiber predominance with only a few very small, scattered type 2A fibers and no type 2B fibers. The small type 2A fibers were frequently hybrid fibers with either slow MyHC or embryonic MyHC expression. The type 1 fibers showed variation in fiber size, internal nuclei and some structural alterations. There was fatty infiltration, which was also demonstrated by MRI.

**Conclusion:**

Dominantly inherited MyHC IIa myopathy due to a splice defect causing loss of amino acids 1860–1891 in the distal tail of the MyHC IIa protein including part of the assembly competence domain. The myopathy is manifesting with slowly progressive muscle weakness without overt ophthalmoplegia and markedly reduced number and size of type 2 fibers.

**Supplementary Information:**

The online version contains supplementary material available at 10.1186/s12883-022-02935-4.

## Introduction

Myosin IIa myopathies are autosomal dominant or recessive disorders, caused by variants in the *MYH2* gene that encodes the fast IIa myosin heavy chain [1]. Myopathy associated with recessive *MYH2* variants is rare but more frequent than the dominantly inherited myosin IIa myopathy. The first patients with recessive myosin IIa myopathy carried compound heterozygous or homozygous truncating variants in *MYH2* [2]. Several additional cases have later been reported carrying either truncating or missense variants [3–10]. All described individuals so far have had external ophthalmoplegia and most have had ptosis and facial muscle weakness, in addition to usually proximal muscle weakness.

Dominantly inherited myosin IIa myopathy appears to be very rare and was first identified in a large Swedish pedigree with numerous affected individuals and associated with a heterozygous missense variant in *MYH2* [11, 12]. Only two additional sporadic cases with de novo heterozygous missense *MYH2* variants have been reported with clinical manifestations, genetics and muscle pathology [13, 14].

We have now identified an additional family with myopathy due to a heterozygous splice-site variant in *MYH2*, which segregated with the muscle disease in the family. We demonstrated abnormal splicing and expression of *MYH2* mRNA lacking exon 39 by cDNA analysis and severe pathological alterations of type 2A muscle fibers. The novel dominant pathogenic *MYH2* variant was associated with a phenotype showing muscle pathology typical for myosin IIa myopathy but lacking the otherwise characteristic ophthalmoplegia.

## Material and methods

**Fig. 1:**
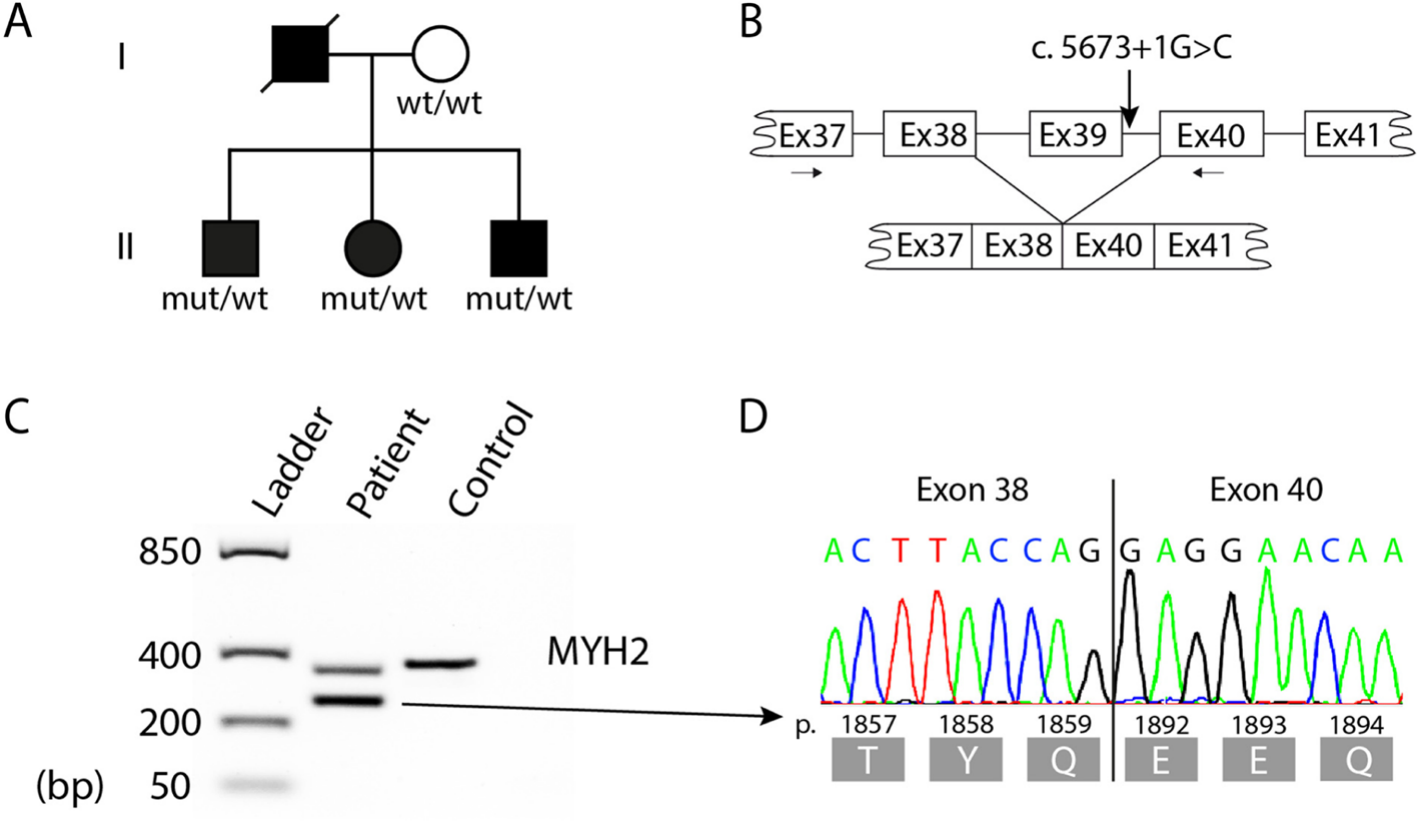
Pedigree and molecular genetics. **A** Pedigree of the family, filled symbols indicate affected individuals and result from the genetic analysis for the variant c.5673 + 1G > C is shown (mut = mutation present, wt = wild type). **B** Schematic illustration showing the splicing effect of the c.5673 + 1G > C variant, which was investigated by reverse transcriptase polymerase chain reaction (RT-PCR). **C** PCR-products from the RT-PCR by gel electrophoresis. In the patient, a strong band with lower molecular size was identified compared to a control sample. **D** Sanger sequencing of cDNA derived from mRNA extracted from skeletal muscle, showing transcript lacking exon 39 (p.1860_1891del)

The pedigree of the family is illustrated in Fig. 1A.

### Case descriptions

Case I:1 This man, who died at age 75 years, was reported to have adult-onset progressive muscle weakness with walking difficulties that severely affected ambulation.

Case I:2 This now 83-year-old woman has had no signs or symptoms of muscle weakness.

Case II:1 This 61-year-old man had progressive muscle weakness starting in his lower limbs at around 20 years of age. He was investigated with muscle biopsy at age 24 years (see below). He is now, at age 61 years, dependent on walking sticks for ambulation. At investigation at age 61 years, he had a mainly proximal muscle weakness affecting hip flexion, knee extension and knee flexion. In the upper extremities he was weak in shoulder abduction, biceps and triceps muscles. He had suffered from neck flexion weakness for many years. There were no signs of sensory impairment. He had ptosis but no ophthalmoplegia. Creatine kinase (CK) was slightly elevated.

Case II:2 This 56-year-old woman experienced slowly progressive muscle weakness from around age 25 years with problems rising from squatting position and climbing stairs. She has not been able to run since age 30 years. From age 47 years, she has been treated for seropositive rheumatoid arthritis, and she had right-sided hearing loss from age 52 years. At investigation at age 56 years, she had difficulties walking uphill and needs support to climb stairs. She had a mainly proximal muscle weakness affecting hip and knee flexion. In the upper extremities, she was weak in shoulder abduction and the triceps muscle. She also had severe neck flexion weakness and slight weakness in wrists and ankles. She had neither ptosis nor ophthalmoplegia. CK and myoglobin were slightly elevated. Muscle biopsy was performed at age 47 years and muscle magnetic resonance imaging (MRI) was performed at age 56 years (see below).

Case II:3 This 54-year-old man experienced muscle weakness affecting the thighs from around 18 years of age. He is able to climb stairs only with difficulties because of leg weakness and falls frequently. At investigation at age 54 years, he had mainly proximal muscle weakness affecting hip flexion, knee extension and, to a minor degree, knee flexion. He showed a positive Gowers’ sign. In the upper extremities he was weak in shoulder abduction, biceps and triceps muscles. He also showed signs of mild distal muscle weakness. There were no signs of sensory impairment. He had neither ptosis nor ophthalmoplegia. CK and myoglobin were slightly elevated.

### Molecular genetics

For genetic analysis, total genomic DNA was isolated from the muscle biopsy specimens from patient II:2 using standard protocols. The DNA was subjected to whole-genome sequencing (WGS) using the TruSeq™ PCR-Free library preparation kit (Illumina, San Diego, CA) and Illumina NovaSeq 6000 platform was used for sequencing (Illumina). The paired-end reads from the WGS were aligned to the reference genome (hg19) and variants were called and filtered for identification of potentially pathogenic variants in candidate genes associated with myopathy.

For RNA studies, total RNA was isolated from frozen skeletal muscle from patient II:2 using the RNeasy Fibrous Tissue Mini Kit (Qiagen, Valencia, CA). RNA was reverse transcribed with the QuantiTect reverse transcription kit (Qiagen), and cDNA was analyzed by PCR and Sanger sequencing. The forward and reverse primers were designed to hybridize to different exons that were separated by large introns to generate a specific PCR-product on cDNA (Fig. 1B).

### Muscle biopsy

Muscle biopsy was performed in case II:1 (deltoid muscle) and case II:2 (vastus lateralis muscle) by open biopsy. Specimens were snap-frozen in isopentane chilled with liquid nitrogen for cryostat sectioning and histochemistry. Standard techniques were used for enzyme histochemistry [15]. Muscle fiber types were assessed by myosin heavy chain (MyHC) by applying quadruple immunofluorescence and scanning in a Hamamatsu S60 digital scanner with fluorescence equipment, including a DAPI/FITC/TRITC/Cy5 quad-band filter set (Semrock, New York). The tissue sections were processed in a Dako Autostainer. Primary antibodies were for slow/beta cardiac MyHC: BA-D5 (mouse IgG2b, DSHB, 1:50), for MyHC IIa: SC-71(mouse IgG1, DSHB, 1:50), for MyHC IIx: 6H1 (mouse IgM, DSHB, 1:10) and for perlecan (basement membranes): Anti-Heparan Sulfate Proteoglycan, MAB1948P (rat IgG2a, cloneA7L6, Merck, 1:200). Secondary antibodies were BV421 (goat-anti-mouse IgG2b, Jackson Immunoresearch Laboratories, 1:200), Alexa Fluor488 (goat anti-mouse IGg1, Invitrogen, 1:200), Alexa Fluor 647 (goat anti-mouse IgM, Invitrogen, 1:200) and Alexa 568 (goat anti-rat IgG, Invitrogen, 1:200).

For other immunostainings brightfield microscopy was applied and tissue sections were processed in a Dako Autostainer using the EnVision FLEX DAB+ Substrate Chromogen System kit and incubated with the following primary antibodies for one hour: anti-embryonic MyHC, F1.652 (DSHB, 1:20) and anti-fetal MyHC, MHn (Leica, 1:20).

### Magnetic resonance imaging

In case II:2, whole-body MRI was performed on an Ingenia 3 T scanner (Philips, Eindhoven, The Netherlands) with a protocol comprising 5 mm thick with 1 mm interslice gap axial l T1-weighted and short tau inversion recovery (STIR) sequences, and coronal STIR sequences.

## Results

### Molecular genetics

Whole-genome sequencing disclosed a heterozygous variant in the MyHC IIa gene (*MYH2*) located in the donor splice-site between exon 39 and intron 39, c.5673 + 1G > C (NM_017534.6) (Fig. 1A, B). The variant was predicted to abolish normal splicing and was not identified in the gnomAD database and had a CADD-score of 34 (CADD: Combined Annotation-Dependent Depletion; https://cadd.gs.washington.edu/). Other candidate genes associated with myopathies according to NMD Gene Table 2022 (www.musclegenetable.fr/) were excluded. Most variants called after filtering were predicted to be benign or with uncertain significance according to computed ACMG Guidelines classification. Sanger sequencing, which was performed for verification and segregation analysis of other family members, revealed that both affected brothers (II:1 and II:3) carried the same splice-site variant in *MYH2* as the index patient (II:2), whereas their mother (case I:2) did not carry the variant implying that in was inherited from their now deceased father (case I:1) who was reported to have had severe muscle weakness.

To analyze the effect of the c.5673 + 1G > C variant on splicing we performed RT-PCR using primers flanking exon 39 (F-primer in exon 37 and R-primer in exon 40 (Fig. 1B) resulting in a PCR product corresponding to a transcript lacking exon 39 (Fig. 1C, D). This result confirms that aberrant splicing is introduced by the variant c.5673 + 1G > C (r.5578_5673del) and transcripts lacking exon 39 are in-frame and are predicted to result in aberrant protein lacking 32 amino acids (p.Thr1860_Ala1891del).

### Muscle biopsy

**Fig. 2:**
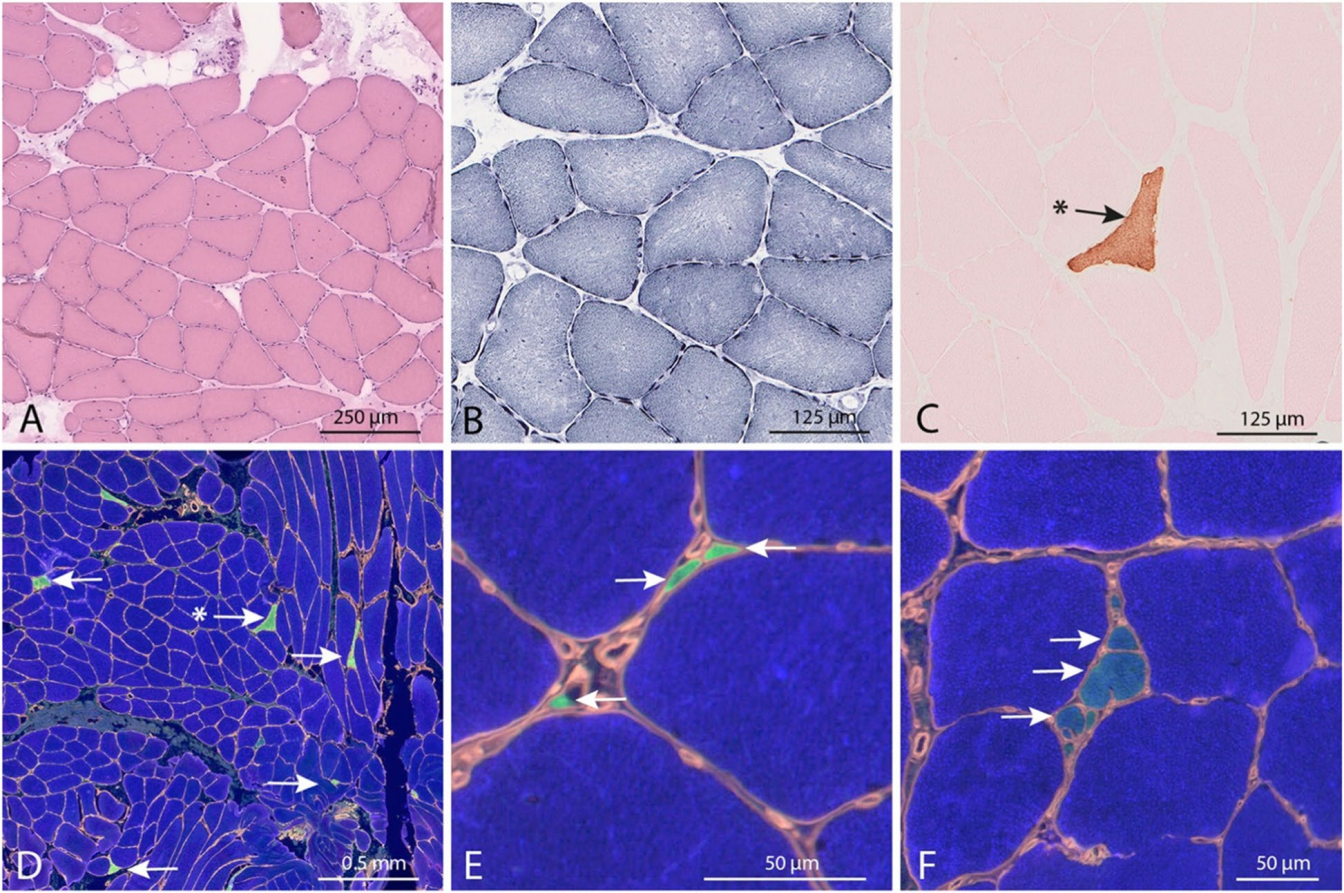
Muscle biopsy from the deltoid muscle in case II:1 at age 24 years. **A** The majority of muscle fibers are larger than normal and many of them show internal nuclei. There are also scattered very small fibers (H&E). **B** The internal structure of the large fibers is slightly disturbed (NADH-TR). **C** Scattered regenerating fibers (arrow) (Immunostaining of embryonic MyHC). **D**-**F** Immunostaining of MyHC isoforms and perlecan; Slow/beta cardiac MyHC: blue, IIa MyHC: green, IIx MyHC: red and perlecan yellow, and for normal control, see Fig. 3F. **D** There is nearly type 1 fiber (blue) uniformity with a few scattered very small type 2A fibers (green, arrows). Some of these express also embryonic MyHC (asterisk in C and D). There are no type 2B fibers. **E** High magnification shows numerous extremely small type 2A fibers (arrows). **F** Some small fibers are hybrids, expressing both slow/beta cardiac MyHC and IIa MyHC (arrows)

**Fig. 3:**
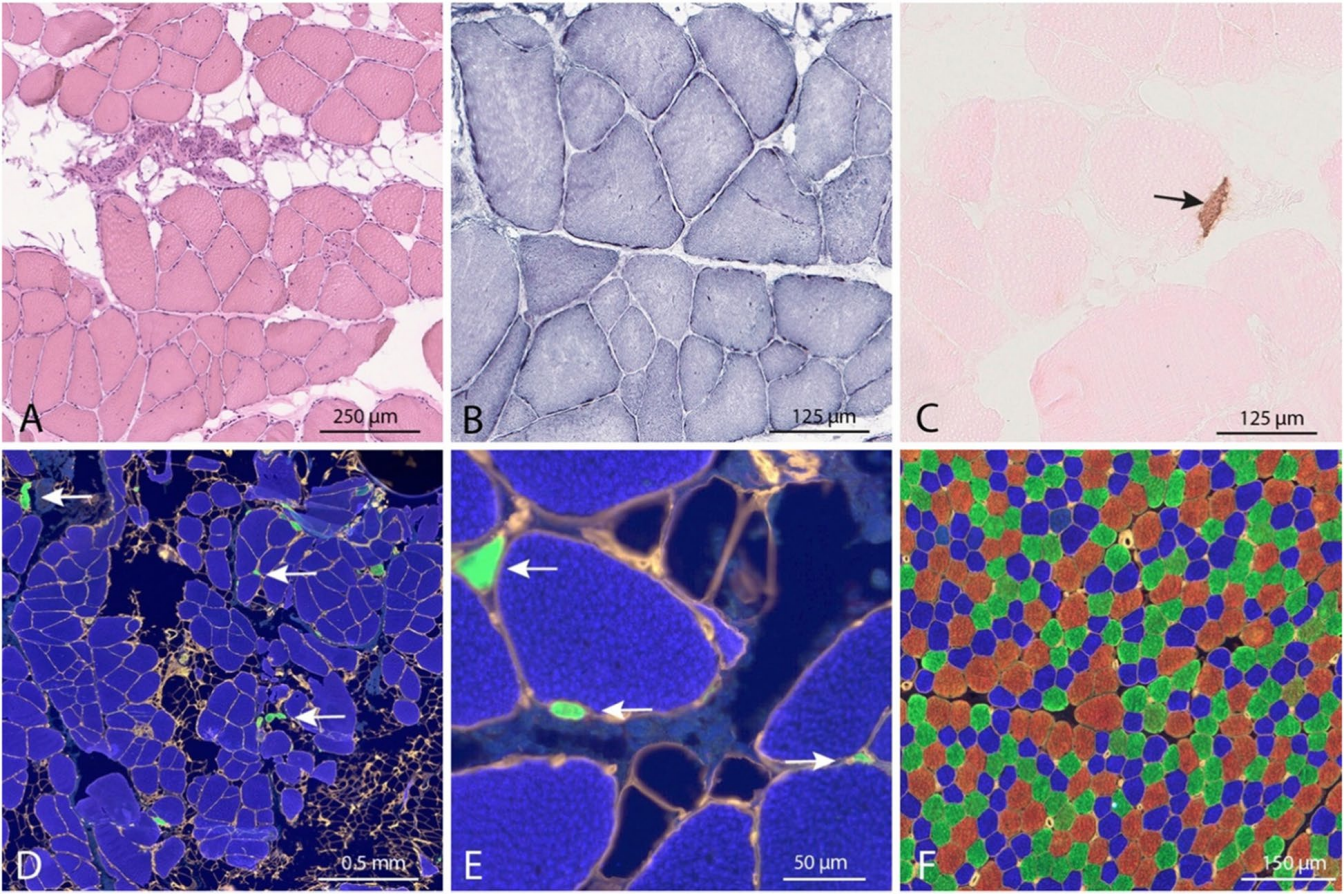
Muscle biopsy from the vastus lateralis muscle in case II:2 at age 55 years. **A** The majority of muscle fibers are larger than normal and many of them show internal nuclei. There are also scattered small and very small fibers, and fatty replacement of muscle fibers (H&E). **B** The internal structure of the large fibers is slightly disturbed (NADH-TR). **C** Scattered regenerating fibers (arrow) (Immunostaining of embryonic MyHC). **D**-**F** Immunostaining of MyHC isoforms and perlecan. For normal control, see **F**. **D** There is nearly type 1 fiber (blue) uniformity with a few scattered very small type 2A fibers (green, arrows). There are no type 2B fibers. **E** High magnification shows numerous extremely small type 2A fibers (arrows). **F** Control muscle from a child without muscle disease demonstrating a normal mosaic pattern of three different fiber types: Type 1 fibers with slow/beta cardiac MyHC: blue, type 2A fibers with MyHC IIa: green, type 2B fibers with MyHC IIx: red and perlecan for basement membranes: yellow

In case II:1, a muscle biopsy at age 24 years from the deltoid muscle revealed muscle fibers that were generally larger than normal and a few extremely small fibers (Fig. 2A). The intermyofibrillar network was regular in most large fibers (Fig. 2B). There was nearly type 1 fiber uniformity, but the scattered extremely small fibers expressed type IIa MyHC (Fig. 2D-F). Some small fibers co-expressed types IIa and embryonic MyHC (Fig. 2C and E). A few small fibers were hybrids, expressing both slow/beta cardiac and IIa MyHC (Fig. 2E). There was no expression of type IIx MyHC. For normal control, see Fig. 3F.

In case II:2 a muscle biopsy at age 55 years from the vastus lateralis muscle revealed marked variability of fiber size with many fibers larger than normal and many atrophic or hypoplastic fibers (Fig. 3A). There were frequent fibers with internalized nuclei and there was also fatty infiltration. The intermyofibrillar network was regular in most fibers, but some split fibers were present (Fig. 3B). Occasional fibers expressed embryonic MyHC (Fig. 3C). There was nearly type 1 fiber uniformity but scattered very small fibers expressed type IIa MyHC (Fig. 3D, E). There was no expression of type IIx MyHC. For normal control, see Fig. 3F.

### Magnetic resonance imaging

**Fig. 4:**
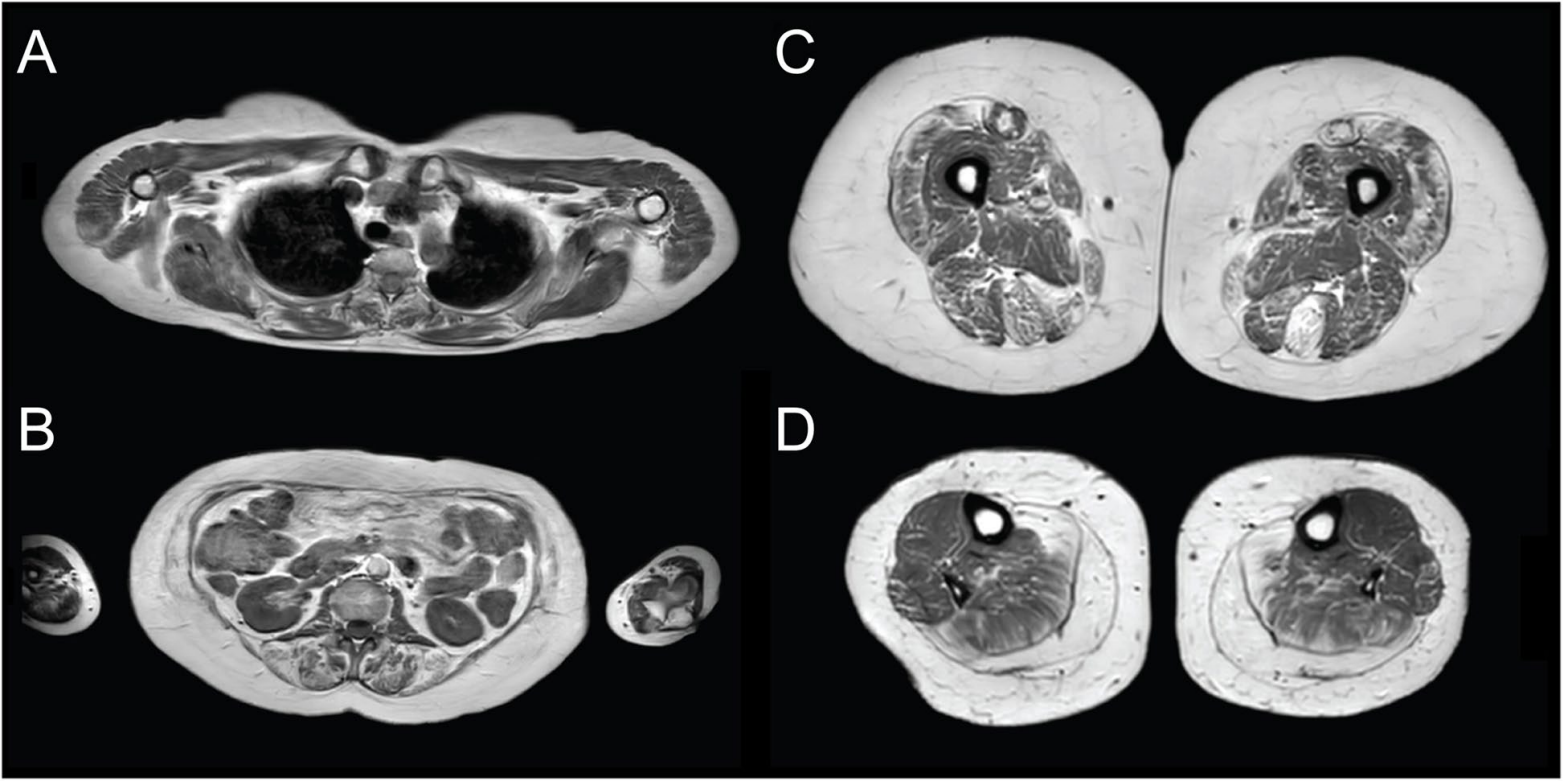
Muscle Magnetic resonance imaging (T1-weighted sequences). **A** Preserved muscles in the shoulder girdle. **B** Severe fatty infiltration of the rectus abdominis and oblique abdominal muscles. **C** Moderate to major degree of fatty infiltration in all muscle groups in the thigh with the exception of the biceps femoris muscle. **D** In the lower leg, both heads of the gastrocnemius muscle show nearly complete replacement by fat tissue and the soleus muscle shows a moderate degree of fatty infiltration whereas the anterior muscle groups are relatively spared

MRI showed extensive fatty infiltration of varying degrees in nearly all muscle groups on the T1-weighted sequences (Fig. 4A-D). Some muscles were, however, almost entirely preserved, such as the shoulder girdle (rotator cuff, deltoid, and pectoralis muscles) except for the subscapularis muscle. The rectus abdominis and oblique abdominal muscles were almost entirely degenerated, whereas the gluteal muscles showed only minor fatty infiltration. Almost the entire thigh musculature was afflicted to a moderate or major degree, with the exception of the biceps femoris muscle. In the lower leg, both heads of the gastrocnemius muscle showed almost complete replacement by fat tissue and the soleus muscle to a moderate degree. However, the anterior muscle groups were almost normal. There were no areas on STIR imaging with increased signal indicating active inflammation.

## Discussion

**Fig. 5:**
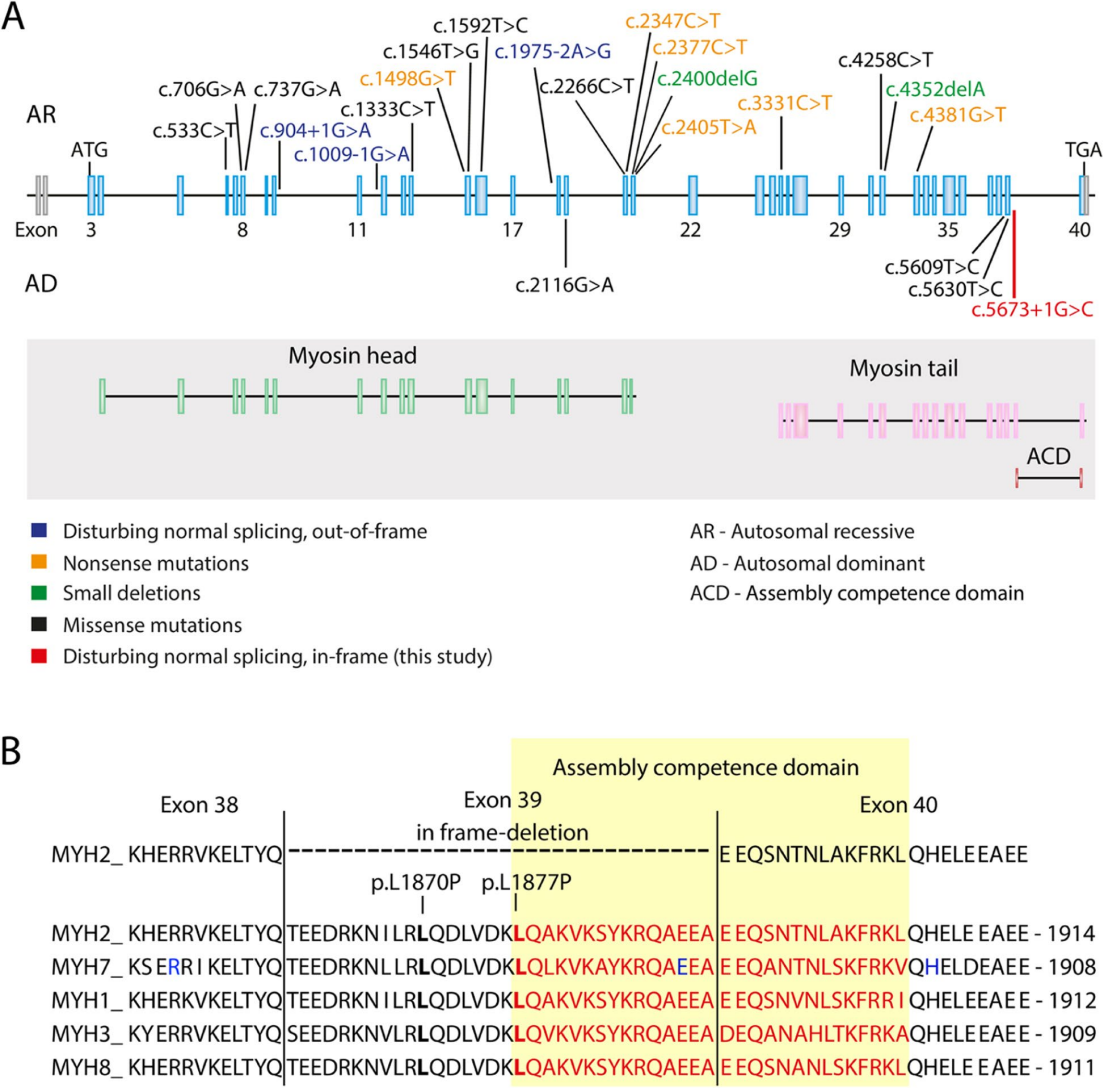
The *MYH2* gene and protein. **A** Schematic illustration of the coding and noncoding regions of the *MYH2* (NM_017534.6), including the novel splice-site variant identified in this study (in red, c.5673 + 1G > C) and previously reported variants. A schematic illustration of the MyHC IIa protein shows the borders between the myosin head and tail domains as well as the assembly competence domain. **B** Human skeletal muscle MyHC isoforms show marked homology. The assembly competence domain (ACD, 29 residues) is marked in red. The aberrant splicing due to the c.5673 + 1G > C variant results in transcript lacking exon 39. These transcripts have a disrupted assembly competence domain by lack of 15 residues of the domain (aa1877–1891). The two other described dominant variants located in this region are marked in bold (c.5609 T > C; p.L1870P and c.5630 T > C; p.L1877P). For *MYH7*, three amino acids causing myosin storage myopathy are marked in blue. One of these is located in the middle of the ACD

Myosin myopathies are caused by dominant or recessive variants in genes encoding myosin heavy or light chains [1]. Most common are myopathies associated with variants in *MYH7*, encoding slow/beta cardiac MyHC, which is expressed in type 1 muscle fibers. No human skeletal myopathy has so far been described to be caused by variants in *MYH1*, encoding fast, type 2X MyHC protein, which is expressed in type 2B fibers. *MYH2* variants are usually recessive and, in most cases, loss-of-function variants causing complete loss of MyHC IIa and therefore associated with absence of type 2A muscle fibers (Fig. 5A) [2–10].

Only three different dominant *MYH2* pathogenic variants accompanied by clinical and muscle histopathological findings have been described in the literature. The first variants concerns a large family with many affected individuals displaying early onset myopathy due to a missense variant E706K, in the functionally important SH1 alpha helix in the head of the MyHC, which undergoes conformational changes during muscle contraction [12]. Several experimental in vivo and in vitro studies have demonstrated the functional consequences of this variant for muscle contraction [16], which may explain the early intrauterine debut resulting in congenital contractures. However, part of the pathogenesis is loss of muscle fibers and increased interstitial connective tissue [17]. From a clinical point of view, the disease varies between individuals in the family [11]. Several individuals were reported to have had joint contractures at birth, which resolved spontaneously. Others experienced proximal and distal muscle weakness from childhood or later in life. In some cases, the disease was progressive, leading to wheelchair dependance for ambulation in adulthood. As in recessive MyHC IIa myopathy, external ophthalmoplegia of variable degree could be demonstrated in the affected individuals. The histopathological changes varied among individuals, partly because of age at biopsy and investigated muscle. Type 2A fibers, when present, were affected by small fiber size, structural alterations such as mini cores and exhibited rimmed vacuoles in some adults. There were dystrophic changes in some but not all adult individuals.

In addition to the large family with the E706K variant in the head of the MyHC, two additional cases with apparently dominant *MYH2* variants have been described both with de novo missense variants in the filament-forming rod region of the MyHC IIa protein. One of these cases presented with a congenial myopathy with dysphagia, respiratory distress and external ophthalmoplegia [14]. The motor development was slightly delayed, but the myopathy was apparently nonprogressive until the age of 12 years, when the female patient was described in a publication as a case report. Muscle histopathology at age nine months demonstrated mild fibroses, type 1 fiber predominance, and small scattered type 2 fibers. A de novo *MYH2* missense variant, L1870P was identified. The other case was a male, also with a de novo missense variant, L1877P [13]. That case presented with progressive symptoms at the age 16 years with proximal and distal muscle weakness, bulbar symptoms and ophthalmoplegia. He was followed until at least 58 years of age in the report. Several muscle biopsies were performed in different muscles at three different ages (from 20 to 46 years). The main findings were increased variability of fiber size, dystrophic changes, lobulated fibers with cores and type 1 fiber predominance with only a few scattered type 2 fibers.

In our MyHC IIa myopathy family, the second family with dominant inheritance described in the literature, we identified muscle pathological changes in line with what has been described in many recessively inherited cases as well as those few reported with dominant disease-causing variants. Such changes involved a marked type I fiber predominance and only a few scattered small type 2 fibers in addition to increased interstitial connective and fat tissue. There was a complete absence of type 2B fibers which has been found previously in many MyHC IIa myopathies.

The three previously described variants that were associated with dominant MyHC IIa myopathy were all missense variants. Two of these (L1870P and L1877P) were located in the distal rod region of the MyHC where they may possibly influence filament assembly and thereby cause myopathy. The distal rod region of the MyHC includes a 29-residue assembly competence domain (ACD) which is important for filament formation [18, 19]. The aberrant splicing resulting in transcript with loss of exon 39 in our family overlaps to a great extent with the ACD (See Fig. 5B). Since a missense variant in *MYH7* (E1886K; marked with a blue letter in Fig. 5B) in the middle of the ACD has been demonstrated to impair severely filament formation, it may be anticipated that the aberrant splicing in our patients with loss of 15 of the 29 residues in the ACD will have strong functional impact on filament formation [18, 19]. The *MYH7* E1886K variant is associated with myosin storage myopathy (MIM #255160) with accumulation in type 1 muscle fibers of hyalin bodies built up of slow/beta cardiac MyHC that has not assembled into proper thick filaments [1, 20]. Myosin storage myopathy with typical hyalin bodies has only been described in association with *MYH7* variants and is therefore considered a disease of *MYH7* [1]. In our family, it was not possible to identify any hyaline bodies in the few very small type 2A fibers. Therefore, one can only speculate that MyHC IIa myopathy caused by variants affecting the ACD as in our family will result in loss and atrophy of type 2A fibers rather than hyalin body myopathy. The nearly complete loss of type 2A fibers resulting from a perturbed assembly of MyHC IIa thick filaments may also explain the similarities in muscle pathology and MRI findings with the recessive MyHC IIa myopathy caused by complete lack of MyHC IIa expression [2].

Since the clinical presentation of MyHC IIa myopathies is variable, no distinct clinical features can be identified. However, external ophthalmoplegia was a consistent finding in previous reports, and in some cases ptosis was present. In the family presented in this report, external ophthalmoplegia could not be unequivocally demonstrated, and ptosis only in one case. Therefore, it seems that dominantly inherited MyHC IIa myopathy may occur without overt ophthalmoplegia.

## Conclusion

In conclusion, we report on the second family in the literature with dominantly inherited MyHC IIa myopathy. The aberrant splicing is predicted to impair severely thick filament formation in type 2A muscle fibers, which may lead to the observed pathology with only few and atrophic type 2A muscle fibers and fatty infiltration. Lack of ophthalmoplegia in our patients indicate that this sign may not be a consistent finding in dominant MyHC IIa myopathy.

## Supplementary Information

**Additional file 1.**

## Data Availability

The data supporting the findings of this study are available from the corresponding author upon reasonable request.

## Abbreviations

MyHC: Myosin heavy chain
MYH2: *Homo sapiens* myosin heavy chain 2
MYH7: *Homo sapiens* myosin heavy chain 7
CK: Creatine kinase
ACD: Assembly competence domain
WGS: Whole-genome sequencing
CADD score: Combined Annotation-Dependent Depletion score
PCR: Polymerase chain reaction
RT-PCR: Reverse transcription PCR
H&E: Hematoxylin and eosin
NADH-TR: Nicotinamide adenine dinucleotide dehydrogenase-tetrazolium reductase
