# Supplementary material for: Dominantly inherited myosin IIa myopathy caused by aberrant splicing of MYH2
Source: BMC Neurol. 2022 Nov 15;22:428. doi: 10.1186/s12883-022-02935-4 (PMC9664609; doi:10.1186/s12883-022-02935-4)
Supplement: Supplementary file 1 — Additional file 1. [file 12883_2022_2935_MOESM1_ESM.docx]

Supplementary Information file


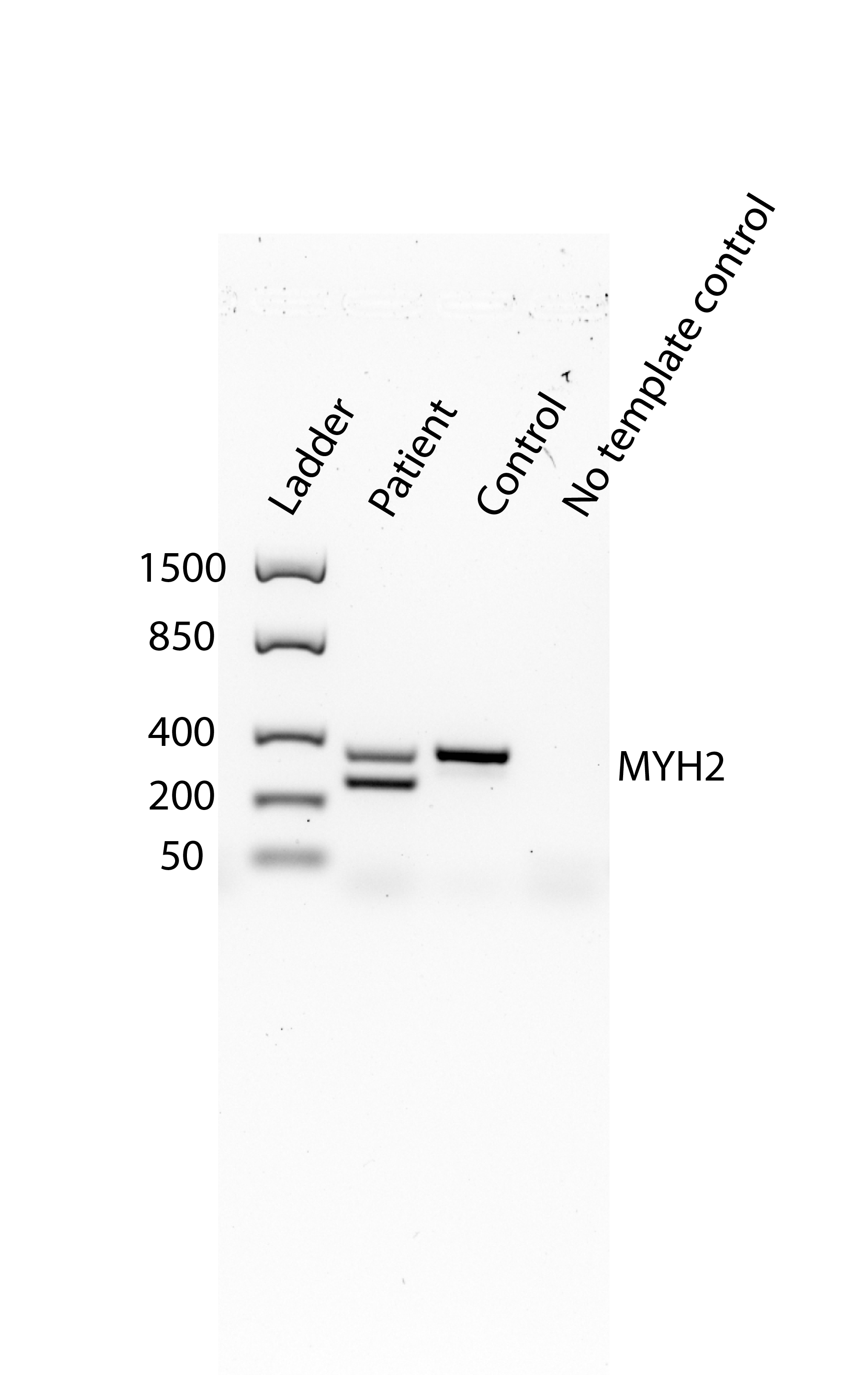


Supplementary figure. Uncropped gel images for “Figure 1C” in the main manuscript file.
